# Impact of a Clinical Valve Coordinator on Hospital Length of Stay and Patient Outcomes: Results From the BENCHMARK Registry

**DOI:** 10.1016/j.shj.2025.100740

**Published:** 2025-10-17

**Authors:** Sandra B. Lauck, Francesco Saia, Eric Durand, Bettina Højberg Kirk, Fiona Kelly, Douglas F. Muir, Gemma McCalmont, Mark S. Spence, Mariuca Vasa-Nicotera, David Wood, Cristóbal A. Urbano Carrillo, Damien Bouchayer, Vlad Anton Iliescu, Christophe Saint Etienne, Nina Fauré, Céline Hee, Florence Leclercq, Vincent Auffret, Lluis Asmarats, Carlo Di Mario, Aurelie Veugeois, Jiri Maly, Andreas Schober, Luis Nombela-Franco, Nikos Werner, Joan Antoni Gómez Hospital, Julia Mascherbauer, Giuseppe Musumeci, Nicolas Meneveau, Thibaud Meurice, Felix Mahfoud, Federico De Marco, Tim Seidler, Florian Leuschner, Patrick Joly, Jean Philippe Collet, Ferdinand Vogt, Emilio Di Lorenzo, Elmar Kuhn, Vicente Peral Disdier, Radka Rakova, Wilbert Wesselink, Jana Kurucova, Violetta Hachaturyan, Claudia M. Lüske, Marie Zielinski, Peter Bramlage, Derk Frank

**Affiliations:** a Centre for Cardiovascular Innovation, University of British Columbia, Vancouver, British Columbia, Canada; b Department of Interventional Cardiology, IRCCS University Hospital of Bologna, Policlinico S. Orsola, Bologna, Italy; c Department of Cardiology, Univ Rouen Normandie, Rouen, France; d Department of Cardiology, Rigshospitalet, Copenhagen University Hospital, Copenhagen, Denmark; e Cardiology Department, Royal Victoria Hospital Belfast, Belfast, Ireland; f Cardiology Department, James Cook University Hospital, Middlesbrough, UK; g Heart and Vascular, Mater Private Network and RCSI University of Medicine & Health Sciences, Dublin, Ireland; h Cardiology Department, Hospital Sindelfingen-Böblingen, Sindelfingen, Germany; i Cardiology Department, Hospital Regional Universitario de Málaga, Malaga, Spain; j Department of Cardiology, The Clinique de l’Infirmerie Protestante, Lyon, France; k Department of Cardiovascular Surgery, University of Medicine and Pharmacy Carol Davila, Bucharest, Romania; l Department of Cardiology, Centre Hospitalier Regional Universitaire de Tours, Hôpital Trousseau, Tours, France; m Cardiology Department, Montpellier University Hospital, Montpellier University, Montpellier, France; n Université de Rennes 1, CHU Rennes Service de Cardiologie, Rennes, France; o Cardiology Department, Hospital de la Santa Creu i Sant Pau, Instituto de Investigación Biomédica Sant Pau, Barcelona, Spain; p Interventional Cardiology Division, Department of Clinical & Experimental Medicine, Careggi University Hospital, Florence, Italy; q Department of Cardiology, Institut Mutualiste Montsouris, Paris, France; r Cardiac Center, IKEM Prague, Prague, Czech Republic; s Department of Cardiology, Hospital Floridsdorf and the Karl Landsteiner Institute for Cardiovascular and Critical Care Research Vienna, Vienna, Austria; t Instituto Cardiovascular, Hospital Clínico San Carlos, Instituto de Investigación Sanitaria del Hospital Clínico San Carlos (IdISSC), Madrid, Spain; u Medical Department III, Heart Center Trier, Krankenhaus der Barmherzigen Brüder, Trier, Germany; v Heart Diseases Institute, Bellvitge University Hospital - IDIBELL, University of Barcelona, L’Hospitalet de Llobregat, Barcelona, Spain; w Department of Internal Medicine 3 / Cardiology, University Hospital St. Pölten, St. Pölten, Austria; x Karl Landsteiner University of Health Sciences, Krems, Austria; y Cardiology Division, AO Ordine Mauriziano, Torino, Italy; z Department of Cardiology, University Hospital Besancon, Besancon, France; aa SINERGIES, University of Burgundy Franche-Comte, Besancon, France; ab Cardiology, Polyclinique Du Bois, Lille, France; ac Department of Cardiology, University Heart Center, University Hospital Basel, Basel, Switzerland; ad Cardiovascular Research Institute Basel (CRIB), University Heart Center, University Hospital Basel, Basel, Switzerland; ae Department of Interventional Cardiology, Centro Cardiologico Monzino IRCCS, Milan, Italy; af Cardiology and Pneumology, University Medical Center Göttingen, Göttingen, Germany; ag Department of Cardiology, Campus Kerckhoff of the Justus-Liebig-Universität Gießen, Kerckhoff-Clinic, Bad Nauheim, Germany; ah Department of Medicine III, University of Heidelberg, German Centre for Cardiovascular Research (DZHK), Germany; ai Department of Interventional Cardiology, Hôpital Saint Joseph, Marseille, France; aj Department de Cardiologie, Hôpital de Pitié-Salpêtrière AP-HP, Paris, France; ak Department of Cardiac Surgery, Artemed Klinikum München Süd, München, Germany; al Department of Cardiac Surgery, Paracelsus Medical University Nuremberg, Germany; am UOC Cardiologia/UTIC, Monaldi Hospital, Neaples, Italy; an Department of Cardiothoracic Surgery, Heart Center, Faculty of Medicine and University Hospital of Cologne, Cologne, Germany; ao Cardiology Department, Hospital Universitari Son Espases, Palma, Spain; ap Grupo de investigación de Fisiopatología y Terapéutica Cardiovascular, Institut d’Investigació Sanitària Illes Balears (IdISBa), Palma, Spain; aq Department of Interventional Cardiology, Edwards Lifesciences, Prague, Czech Republic; ar Institute for Pharmacology and Preventive Medicine, Cloppenburg, Germany; as Department of Internal Medicine III (Cardiology and Critical Care Medicine), University Clinical Centre Schleswig-Holstein (UKSH), Kiel, Germany; at German Centre for Cardiovascular Research, Partner Site Hamburg/Kiel/Lübeck, Kiel, Germany

**Keywords:** Aortic stenosis, Clinical valve coordinator, Quality of care, Transcatheter aortic valve implantation, TAVI

## Abstract

**Background:**

Transcatheter aortic valve implantation (TAVI) treatment pathways can be supported by a dedicated clinical valve coordinator (CVC), enhancing their efficiency. We aimed to evaluate the impact of a CVC in managing the treatment pathway of patients undergoing TAVI across Europe before and after implementing 8 Benchmark best practices.

**Methods:**

The BENCHMARK registry (ClinicalTrials NCT04579445) was a multicenter international study of patients with severe symptomatic aortic stenosis undergoing TAVI with balloon-expandable valves across 28 European centers. Primary outcomes were hospital and intensive care length of stay (LoS). The secondary outcome was 30-day patient safety.

**Results:**

Of 2323 patients, 1262 were treated at centers without a pre-existing CVC and 1061 at centers with a pre-existing CVC; propensity matching resulted in 891 matched pairs. The total procedural time was significantly reduced in both groups (*p* < 0.001) after implementing Benchmark best practices. Hospital LoS was lower before Benchmark when a CVC was present and was significantly shorter in both groups following implementation (*p* < 0.001), as was the critical care LoS (*p* < 0.001). The presence of a CVC did not affect safety outcomes but was associated with a reduced risk of major vascular bleeding when combined with Benchmark best practices. Patient satisfaction was higher in centers with a pre-existing CVC (*p* < 0.001).

**Conclusions:**

The addition of a CVC to the multidisciplinary team and their sustained contributions to processes of care align with the implementation of Benchmark practices, significantly decrease the health service requirements of TAVI patients, and are associated with improved patient-reported experiences.

## Highlights

• A clinical valve coordinator (CVC) in a Benchmark center reduces health service requirements.
• A CVC is linked to a reduced admission time in critical care and a shorter length of stay.
• The presence of a CVC is associated with significantly higher patient satisfaction.

## Introduction

With the rapid expansion of transcatheter aortic valve implantation (TAVI), patient need for timely treatment and referral volumes are increasing, whereas there is growing awareness of the undertreatment of aortic stenosis.^1^^,^^2^ In this context, improving patient pathways and optimizing current practices have become essential to ensure access to care and program efficiency. International efforts are underway to improve patients' journey of care to achieve this goal.^3^ There is growing evidence that the careful consideration and implementation of coordinated screening, minimalist periprocedure approach, facilitated postprocedure recovery, and criteria-driven safe discharge home are required to achieve consistent, efficient, and patient-centered processes across regions.^4^^,^^5^

The treatment of people with aortic stenosis is uniquely complex, with multiple moving parts.^6^ To address these challenges, the role of the clinical valve coordinator (CVC) has emerged as a key member of the multidisciplinary TAVI team and a central point of contact for patients and their families.^7^^,^^8^ The role of the CVC—also known as "TAVI coordinator", "valve clinic coordinator" or other local terms—is most often held by a nurse or advanced practice provider.^9^ Responsibilities focus on the coordination of the patients’ treatment journey, the provision of direct and indirect care, program leadership, and communication pathways.^5^^,^^10^^,^^11^ The role of the CVC was inspired by the requirements of early TAVI pivotal trials and the subsequent successful implementation in North America, the United Kingdom, Australia, and New Zealand.^10^ This has led to the inclusion of this role in valvular heart disease guidelines.^12^ Although their professional preparation may not be specified, most statements imply that the role reaches far beyond administrative or clerical responsibilities and requires clinical competencies.^13^, ^14^, ^15^, ^16^, ^17^ There is emerging evidence that this unique position plays a role in reducing length of stay (LoS), optimizing staffing models, supporting standardized postprocedural processes, and improving patient safety and satisfaction.^6^^,^^18^ However, despite early success and proven impact, the role of the CVC has only recently begun to elicit a surge in interest in European countries and Asia to help with growing patient and program needs.^7^ Furthermore, responsibilities and implementation of the CVC remain inconsistently defined across programs.^19^^,^^20^

There is scarce evidence from prospective, multicenter studies on the contributions of a CVC to outcomes and program efficiencies, especially across multiple European countries. To address this gap, we aimed to evaluate the impact of the CVC on managing the treatment pathway of patients with severe symptomatic aortic stenosis treated with TAVI before and after the implementation of the 8 Benchmark best practices^21^^,^^22^ across Europe.

## Methods

BENCHMARK (ClinicalTrials NCT04579445) was a multicenter international study of patients with severe symptomatic aortic stenosis undergoing TAVI at 28 European centers in Austria, France, Spain, Germany, Italy, Czech Republic, and Romania.^21^^,^^22^ BENCHMARK was conducted according to the European Medical Device Regulations and the Declaration of Helsinki. Ethical approval was obtained from the independent ethics review boards at each participating site. All patients provided written informed consent.

### Patient Selection

All patients included in the present analysis were enrolled in the BENCHMARK registry (N = 2323). Inclusion and exclusion criteria have been previously described.^22^

### Benchmark Best Practices

**Supplementary Figure S1:**
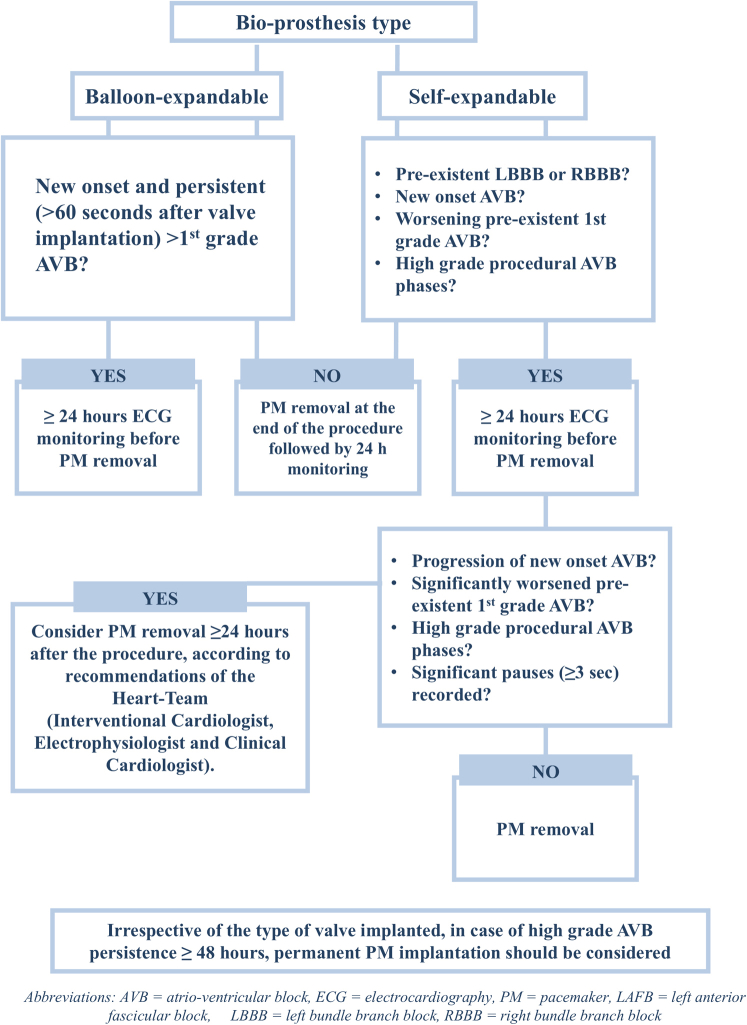
**Decision tree with criteria that encourage early pacemaker removal.**

**Supplementary Figure S2:**
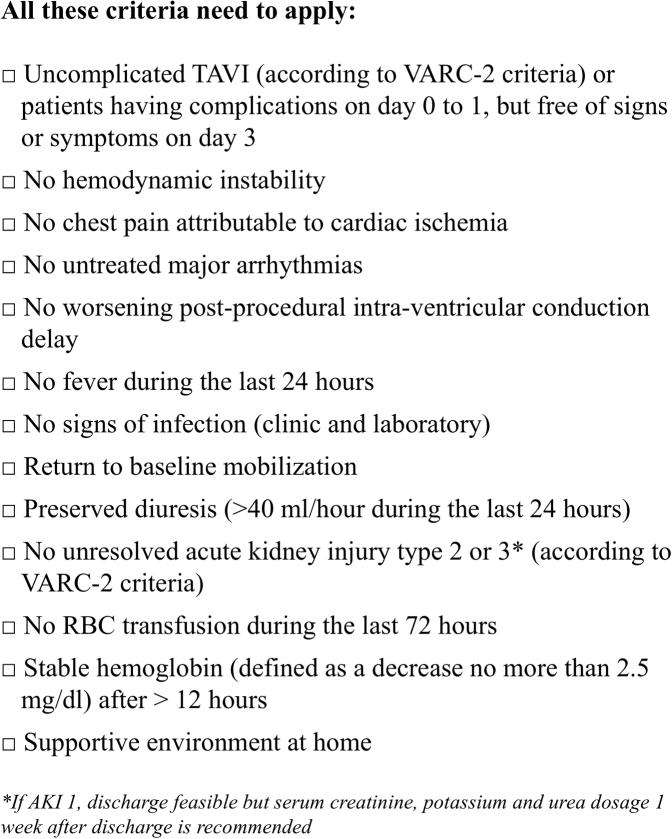
**Checklist for criteria-based discharge encouraging <72 hours discharge.** Abbreviations: AKI, acute kidney injury; RBC, red blood cell; TAVI, transcatheter aortic valve implantation; VARC, Valve Academic Research Consortium.

**Supplementary Figure S3:**
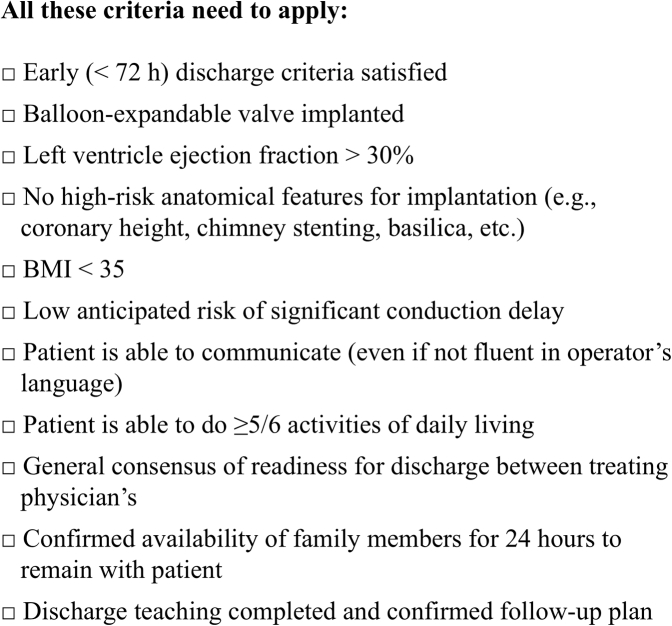
**Checklist for criteria-based discharge encouraging ∼ 24 hours discarge.** Abbreviation: BMI, body mass index.

The Benchmark best practices were developed on the basis of contemporary evidence and expert clinical opinions,^7^ and included targeted quality of care indicators that have been unevenly adopted across diverse European regions: 1) tailored education of patient and family to facilitate early discharge; 2) education and alignment of the internal team to standardize practice; 3) documented anticipated discharge date at admission; 4) echocardiographic or angiographic check at the end of the procedure; 5) early nurse-led and standardized mobilization protocol; 6) decision support tool to determine the need for permanent pacemaker (PPM) implantation (Supplementary Figure 1); 7) daily visit by implanting physician; and 8) criteria-based discharge home (Supplementary Figures 2 and 3). The initiation of the implementation process was preceded by a self-assessment of the hospital’s current alignment with Benchmark, quality of care indicators, and the required training needs. The implementation process of the Benchmark protocol was completed in a 2-month period. Details on the implementation process were previously described elsewhere.^22^

### Role of Clinical Valve Coordinator

The CVC position (nurse coordinator, study nurse, clinical research assistant, or other) was already established in 13 centers (46%), and 14 centers (54%) developed the role at the start of the study as recommended. One site participated without a CVC and was excluded from the present analysis. The study mandated the presence of at least 1 CVC at each participating center. CVCs were recognized as coinvestigators in the study and were responsible for leading the implementation of nursing-focused best practices, contributing to clinical care as appropriate, and collaborating with implanting physicians, clinical teams, and administration to facilitate the change management required for the sustained adoption of the Benchmark best practices.

The CVC leveraged their clinical expertise to facilitate patients’ journey of care from admission to discharge to support the implementation of practice changes. Their direct contact with treating physicians, nursing teams, and other clinicians further enabled them to influence care decisions and the implementation of standard operating procedures and decision support tools (e.g., decisions regarding management of new conduction delay and implantation of PPMs) within their licensed scope of practice. The CVC supported nursing teams to implement changes to the clinical pathway (e.g., monitoring protocols, early mobilization, discharge teaching) and collaborated with medical teams to standardize in-hospital surveillance, criteria-based discharge, and follow-up protocols. The conduct of the trial was facilitated by a peer-to-peer mentorship program that connected Benchmark CVCs to nurse leaders who provided tailored guidance, support, and resources.

### Study Outcomes

Total hospital LoS, duration of critical care stay (intensive care unit, cardiac/coronary care unit or intermediate medical care), patient satisfaction, and patient safety (e.g., vascular injury) after 30 days were assessed after the implementation of Benchmark best practices. Patient-reported satisfaction was measured using an investigator-adapted version of the Short Assessment of Patient Satisfaction questionnaire,^23^ augmented with supplemental questions on patients’ experiences with communication pertinent to TAVI care. Early patient safety was assessed according to Valve Academic Research Consortium 3 criteria.^24^

### Statistical Analysis

Data were analyzed using descriptive statistics, with categorical variables presented as absolute values and frequencies (%) and continuous variables presented as means (SD and/or median (interquartile range). The calculation of percentages was based on the number of patients with valid data per parameter (i.e., excluding patients with missing information). Comparisons were performed using a t-test or Mann–Whitney *U*-test for continuous variables, as appropriate depending on distribution, and a Fisher exact or chi-square test for categorical variables. The Shapiro–Wilk test was used to test for normal distribution. A *p* value <0.05 was considered significant and is presented in bold font in the tables.

Propensity scores were calculated using a generalized linear model to assess the effects of the pre-existence of a CVC position. Covariates that had a *p* value ≤0.1 in the full-cohort comparison were selected to calculate scores; these included the following pertinent variables: severe frailty, hypertension, previous myocardial infarction, dizziness with exertion, diabetes, abnormal ejection fraction, and New York Heart Association functional classification. The matching was performed using nearest neighbor matching with a 0.2 logit caliper of the propensity scores using the R package "Matchit".^25^ Postmatching, standardized mean differences were analyzed for all covariates included in the propensity score calculation with the R package "WeightIt".^26^ The mean differences for all covariates postmatching were within a desirable threshold (±0.1), indicating adequate balance. All statistical analyses were performed using R statistical environment (version 4.3, R Foundation for Statistical Computing, Vienna, Austria).

## Results

### Patient Population

**Figure 1:**
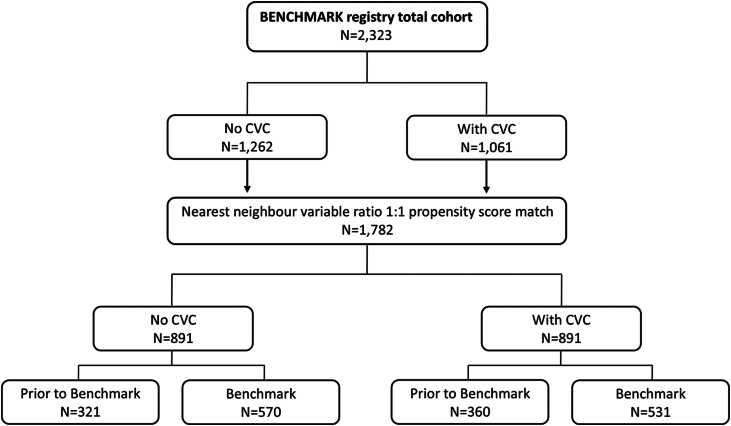
**Study flowchart** Abbreviations: CVC, clinical valve coordinator.

A total of 2323 patients from the BENCHMARK registry were divided into 2 comparative cohorts based on 1) the presence of a pre-existing CVC at the enrolling site before the implementation of Benchmark (reported as no [prior] CVC vs. with [prior] CVC) and 2) by the timing of enrollment (prior to vs. after Benchmark, Figure 1). Overall, 1262 (54.3%) patients were treated at sites without a CVC and 1061 (45.7%) at sites with a pre-existing CVC. Propensity score matching resulted in 891 matched pairs. In centers without a CVC, 321 patients were enrolled before the implementation of Benchmark best practices and 570 after; in centers with a pre-existing CVC, 360 patients were documented before and 531 after Benchmark.

The total unmatched study population revealed several imbalances between the cohorts; after matching, statistically significant differences remained only for body mass index (BMI) (27.2 [no CVC] vs. 28.1 kg/m^2^ [with CVC], *p* < 0.001) and impaired mobility (13.4% [no CVC] vs. 8.2% [with CVC], *p* < 0.001; Table 1).

**Table 1:** Baseline characteristics—unmatched and matched cohorts

Mean ± SD or median (IQR) or n (%)	Unmatched cohort			Matched cohort		
	No CVC (N = 1262)	With CVC (N = 1061)	*p* value	No CVC (N = 891)	With CVC (N = 891)	*p* value
Age (y)	80.0 ± 6.5 81.0 (76.0, 84.0)	79.7 ± 7.1 81.0 (75.0, 85.0)	0.524	80.1 ± 6.4 81.0 (76.0, 85.0)	79.6 ± 7.2 81.0 (75.0, 85.0)	0.197
Female gender	479 (38.0)	427 (40.2)	0.260	328 (36.8)	365 (41.0)	0.072
Body mass index (kg/m^2^)	27.4 ± 5.1 26.7 (24.1, 29.8)	28.0 ± 4.8 27.6 (24.5, 30.7)	**<0.001**	27.2 ± 5.0 26.5 (24.0, 29.7)	28.1 ± 4.8 27.7 (24.6, 30.8)	**<0.001**
AV-related symptoms						
Dizziness with exertion	394 (31.5)	169 (16.0)	**<0.001**	151 (16.9)	167 (18.7)	0.322
(Pre-)syncope	134 (10.7)	105 (10.0)	0.547	80 (9.0)	98 (11.0)	0.155
NYHA class III or IV	731 (58.4)	622 (59.1)	0.737	531 (59.6)	533 (59.8)	0.923
Angina CCS 3 or 4	56 (4.5)	56 (5.3)	0.349	37 (4.2)	51 (5.7)	0.126
Risk scores and frailty						
EuroSCORE II	4.8 ± 5.4 3.2 (1.9, 5.2)	5.0 ± 6.4 2.9 (1.7, 5.7)	0.170	4.8 ± 5.3 3.1 (1.8, 5.4)	4.8 ± 5.6 2.8 (1.7, 5.5)	0.125
Frailty, severe	55 (4.4)	22 (2.1)	**0.002**	22 (2.5)	20 (2.2)	0.755
Impaired mobility	211 (16.8)	87 (8.2)	**<0.001**	119 (13.4)	73 (8.2)	**<0.001**
Cognitive deficit	48 (3.8)	40 (3.8)	0.979	26 (2.9)	33 (3.7)	0.354
Comorbidities						
Previous MI	227 (18.0)	105 (9.9)	**<0.001**	108 (12.1)	97 (10.9)	0.414
Peripheral artery disease	184 (14.6)	168 (15.9)	0.388	131 (14.7)	139 (15.6)	0.597
Diabetes mellitus	447 (35.5)	275 (26.0)	**<0.001**	246 (27.6)	232 (26.0)	0.454
Hypertension	1050 (83.4)	847 (80.2)	**0.047**	717 (80.5)	691 (77.6)	0.130
Renal insufficiency	310 (24.6)	281 (26.5)	0.298	230 (25.8)	233 (26.2)	0.871
Prior PPM	88 (7.0)	89 (8.4)	0.203	66 (7.4)	79 (8.9)	0.266
LVEF <50%	196 (16.0)	203 (19.6)	**0.026**	147 (16.5)	138 (15.5)	0.561
AV block 2nd or 3rd degree	11 (0.9)	16 (1.6)	0.127	7 (0.8)	14 (1.6)	0.103
Creatinine, mg/dl	1.2 ± 0.7 1.0 (0.8, 1.3)	1.3 ± 1.3 1.0 (0.8, 1.3)	0.329	1.1 ± 0.6 1.0 (0.8, 1.3)	1.3 ± 1.1 1.0 (0.8, 1.3)	0.224

Abbreviations: AV, atrioventricular block; CCS, Canadian Cardiovascular Society; CVC, clinical valve coordinator; EuroSCORE, European System for Cardiac Operative Risk Evaluation; IQR, interquartile range; LVEF, left ventricular ejection fraction; MI, myocardial infarction; NYHA, New York Heart Association; PPM, permanent pacemaker.

### Procedural Details

Procedural details for the matched patients are provided in Table 2. Conscious sedation was more frequently used in patients treated after Benchmark implementation in centers with a CVC present before the study (no CVC: 95.4 vs. 92.5%, *p* = 0.072; with CVC: 97.9 vs. 79.2%, *p* < 0.001). The adoption of lighter sedation protocols, defined as local anesthesia with or without minimal sedation combined with anxiolytics or local anesthesia with moderate sedation, following program implementation increased significantly in both cohorts (*p* < 0.001). The total procedure time (from first puncture to sheath removal/closure) was significantly reduced after the implementation of Benchmark regardless of whether a CVC had been previously present (median 52.0 vs. 45.0 min, *p* < 0.001) or not (median 60.0 vs. 45.0 min, *p* < 0.001); there was a significant reduction in the intervention time (from start of sedation to exit of the procedure room) observed at centers with no pre-existing CVC (median 90.0 vs. 75.0 min, *p* = 0.003) but not when the CVC role was established (median 98.0 vs. 95.0 min, *p* = 0.061).

**Table 2:** Procedural details—matched cohort

Mean ± SD or median (IQR) or n (%)	No CVC			With CVC		
	Before Benchmark (N = 321)	Benchmark (N = 570)	*p*-value	Before Benchmark (N = 360)	Benchmark (N = 531)	*p* value
Conscious sedation	297 (92.5)	542 (95.4)	0.072	285 (79.2)	520 (97.9)	**<0.001**
Sedation method			**<0.001**			**<0.001**
LA w/o sedation/anxiolytic	25 (8.4)	38 (7.1)		24 (8.4)	82 (15.8)	
LA + minimal sedation/anxiolytic	110 (37.0)	178 (33.0)		199 (69.8)	291 (56.0)	
LA + moderate sedation	118 (39.7)	282 (52.3)		29 (10.2)	135 (26.0)	
Deep sedation (sleep throughout most of the procedure)	44 (14.8)	41 (7.6)		33 (11.6)	12 (2.3)	
Implanted valve			**<0.001**			**<0.001**
Sapien 3	199 (62.0)	275 (48.4)		299 (83.1)	376 (70.8)	
Sapien 3 Ultra	122 (38.0)	292 (51.4)		61 (16.9)	154 (29.0)	
Total procedure time	67.8 ± 34.0 60.0 (45.0, 90.0)	53.1 ± 29.1 45.0 (33.0, 65.0)	**<0.001**	61.0 ± 34.2 52.0 (37.0, 77.0)	53.0 ± 29.5 45.0 (30.0, 65.0)	**<0.001**
Intervention time (from start of sedation to exit of operating room)	89.9 ± 38.7 90.0 (60.0, 119.0)	82.2 ± 36.7 75.0 (55.0, 100.0)	**0.003**	99.6 ± 45.6 98.0 (65.3, 125.0)	93.3 ± 42.0 95.0 (55.0, 124.0)	0.061
Procedural success						
Absence of procedural mortality	321 (100.0)	563 (99.6)	0.537	360 (100.0)	529 (99.8)	1.000
Correct positioning of valve	321 (100.0)	563 (99.6)	0.537	360 (100.0)	530 (100.0)	1.000
Intended performance of valve	317 (99.4)	557 (98.6)	0.344	359 (99.7)	527 (99.4)	0.651
Device malfunction	1 (0.3)	1 (0.2)	1.000	1 (0.3)	0 (0.0)	0.404
Complications						
Permanent Pacemaker implanted	17 (5.3)	32 (5.6)	0.832	42 (11.7)	33 (6.2)	**0.004**
Conversion to conventional surgery	1 (0.3)	5 (0.9)	0.424	4 (1.1)	0 (0.0)	**0.026**
Bleeding	8 (2.5)	9 (1.6)	0.363	17 (4.7)	13 (2.4)	0.062

Abbreviations: CVC, clinical valve coordinator; IQR, interquartile range; LA, local anesthesia.

### Length of Stay and Critical Care Admission

**Figure 2:**
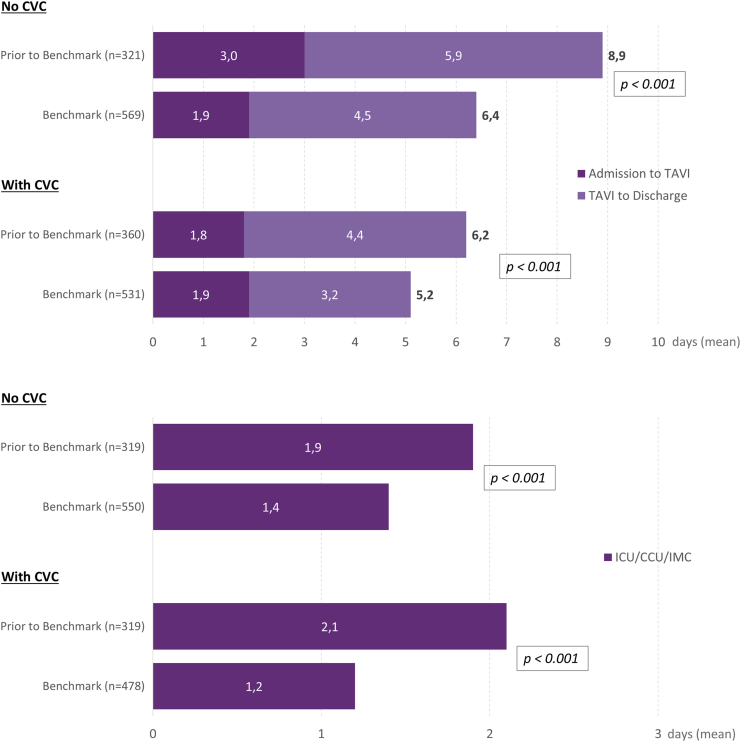
**Total hospital LoS (upper panel) and LoS in critical care (ICU, CCU, IMC; lower panel)—matched cohort** Abbreviations: CCU, coronary care unit; CVC, clinical valve coordinator; ICU, intensive care unit; IMC, intermediate care; LoS, length of stay; TAVI, transcatheter aortic valve implantation.

Total hospital LoS (admission to discharge) in patients treated in centers with no pre-existing CVC was significantly reduced after the implementation of Benchmark (mean 8.9 vs. 6.4 days, *p* < 0.001; Figure 2, Table 3). In centers where the CVC role was previously adopted, the total LoS was significantly lower at baseline and was decreased following implementation (mean 6.2 vs. 5.2 days, *p* < 0.001).

**Table 3:** Length of stay—matched cohort

Mean ± SD	No CVC			With CVC		
	Before Benchmark (N = 319)	Benchmark (N = 550)	*p* value	Before Benchmark (N = 319)	Benchmark (N = 478)	*p* value
Pre-TAVI stay, d	3.0 ± 5.9	1.9 ± 4.1	**0.005**	1.8 ± 2.5	1.9 ± 3.2	0.811
ICU stay, d	0.4 ± 0.8	0.4 ± 0.9	0.858	0.8 ± 1.3	0.5 ± 0.9	**0.001**
CCU stay, d	0.8 ± 2.0	0.4 ± 1.5	**<0.001**	0.6 ± 1.2	0.3 ± 0.9	**<0.001**
IMC stay, d	0.7 ± 2.5	0.6 ± 1.5	0.763	0.7 ± 1.4	0.4 ± 1.1	**<0.001**
GW stay	4.1 ± 5.9	3.3 ± 4.8	**0.005**	2.2 ± 2.4	2.1 ± 3.2	0.598
Post-TAVI stay, d	5.9 ± 6.3	4.5 ± 4.4	**<0.001**	4.4 ± 2.4	3.2 ± 2.3	**<0.001**
Total stay, d	8.9 ± 8.7	6.4 ± 6.5	**<0.001**	6.2 ± 3.7	5.2 ± 4.3	**<0.001**

Abbreviations: CCU, coronary care unit; CVC, Clinical Valve Coordinator; ICU, intensive care unit; IMC, intermediate care unit; GW, general ward; TAVI, transcatheter aortic valve replacement.

The duration of post-TAVI admission to critical care in centers was significantly reduced in both cohorts; centers with a pre-existing CVC achieved a lower overall critical care admission time (mean 2.1 vs. 1.2 days; median 1.2 vs. 0.9 days; *p* < 0.001) compared to centers with no pre-existing CVC (mean 1.9 vs. 1.4 days; median 1.0 vs. 0.9 days; *p* < 0.001; Figure 2).

Patients were more likely to be discharged home the next day in centers after Benchmark implementation regardless of a pre-existing CVC (14.2 vs. 0.3%, *p* < 0.001) or not (13.6 vs. 1.7%, *p* < 0.001; Table 4).

**Table 4:** Discharge details—matched cohort

n (%)	No CVC			With CVC		
	Before Benchmark (N = 319)	Benchmark (N = 550)	*p* value	Before Benchmark (N = 319)	Benchmark (N = 478)	*p* value
Discharge to			0.263			**0.034**
Home	286 (89.7)	509 (92.5)		293 (91.8)	458 (95.8)	
Another hospital	23 (7.2)	22 (4.0)		15 (4.7)	10 (2.1)	
Short-term care	2 (0.6)	2 (0.4)		3 (0.9)	3 (0.6)	
Rehabilitation	7 (2.2)	11 (2.0)		8 (2.5)	4 (0.8)	
Permanent nursing facility	0 (0.0)	3 (0.5)		0 (0.0)	0 (0.0)	
Death	1 (0.3)	3 (0.5)		0 (0.0)	3 (0.6)	
Next-day discharge (home)	5 (1.7)	69 (13.6)	**<0.001**	1 (0.3)	65 (14.2)	**<0.001**

Abbreviations: CVC, clinical valve coordinator.

### Thirty-Day Outcomes

There were no differences in 30-day mortality, stroke/transient ischemic attack, life-threatening bleeding, acute kidney injury, and coronary artery obstruction requiring intervention, irrespective of the presence of a CVC in the 2 cohorts (Table 5). Moreover, the rates of PPM implantation were lower for all patients after the implementation of Benchmark (no CVC: 20.1 vs. 12.3%, *p* = 0.003; with CVC: 19.2 vs. 12.3%, *p* = 0.008). Rates of major vascular complications were reduced after the implementation of Benchmark in centers with a pre-existing CVC (4.5 vs. 1.9%, *p* = 0.029).

**Table 5:** Thirty-day outcomes—matched cohort

n (%)	No CVC			With CVC		
	Before Benchmark (N = 321)	Benchmark (N = 570)	*p* value	Before Benchmark (N = 360)	Benchmark (N = 531)	*p* value
All-cause mortality	1 (0.3)	3 (0.6)	1.000	0 (0.0)	3 (0.6)	0.282
Stroke/TIA	5 (1.7)	7 (1.5)	1.000	1 (0.3)	6 (1.2)	0.258
Life-threatening bleeding	4 (1.3)	6 (1.3)	1.000	5 (1.6)	5 (1.0)	0.522
AKI (stage 2/3, incl. dialysis)	1 (0.3)	4 (0.8)	0.654	1 (0.3)	3 (0.6)	1.000
Coronary artery obstruction requiring intervention	0 (0.0)	1 (0.2)	1.000	4 (1.3)	4 (0.8)	0.718
Major vascular complication	9 (3.0)	12 (2.5)	0.701	14 (4.5)	9 (1.9)	**0.029**
PPI	61 (20.1)	59 (12.3)	**0.003**	60 (19.2)	60 (12.3)	**0.008**
Rehospitalization (all-cause)	25 (8.3)	26 (5.5)	0.127	13 (4.2)	29 (6.0)	0.283

Abbreviations: AKI, acute kidney injury; CVC, clinical valve coordinator; PPI, permanent pacemaker implantation; TIA, transient ischemic attack.

### Patient Satisfaction

**Figure 3:**
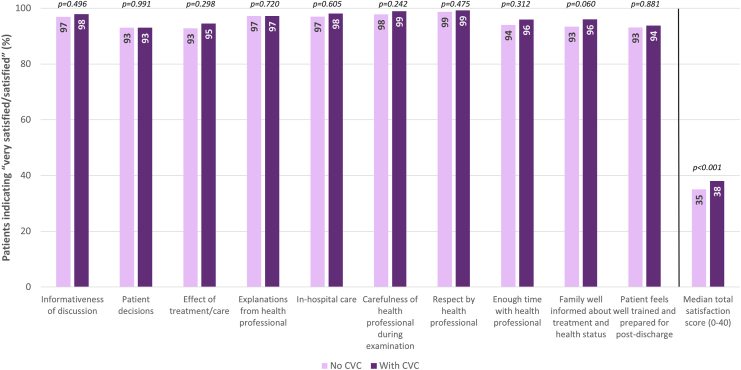
**Patient satisfaction after Benchmark implementation** Abbreviations: CVC, clinical valve coordinator.

Although all patients reported high satisfaction (>90%) in all domains after Benchmark implementation, regardless of the presence of a CVC (Figure 3), the overall patient satisfaction score was significantly higher in centers with a pre-existing CVC (38 vs. 35 points, *p* < 0.001).

## Discussion

The current study aimed to parse the effect of the CVC—a unique role that combines clinical assessment, patient navigation, coordination, and program leadership—to strengthen evidence for informing the care of patients with valvular heart disease and optimizing TAVI programs. We demonstrated that the CVC role amplified the impact of the Benchmark program, as reflected in the significant reduction in total hospital LoS and critical care unit LoS, the improvement in patient satisfaction, and the improvement of 30-day safety as measured by a decreased risk of major vascular bleeding. This novel evidence provides further guidance to support the journey of care of patients with valvular heart disease and to accelerate the adoption of the CVC role across TAVI programs.

### Contemporary TAVI: Changing the Way We Care for Patients

TAVI has become a highly predictable procedure with excellent outcomes across regions and has surpassed surgical aortic valve replacement as the dominant procedure.^27^ However, there is significant heterogeneity in processes of care and clinical pathways, resulting in variation in procedural practices, postprocedure care, and LoS—an indicator of the cumulative impact of the intersecting variables of preprocedure education and planning, clinical practices, and the mitigation of in-hospital practices.^28^ Multiple studies have demonstrated the safety, effectiveness, and cost savings associated with the implementation of a comprehensive bundle of streamlined care for TAVI patients to facilitate early discharge, including multidisciplinary, multimodality, but minimalist transcatheter aortic valve replacement,^29^^,^^30^ FAST-TAVI I and II,^31^^,^^32^ and BENCHMARK.^21^ There is also growing evidence that same-day discharge may be feasible and ideally suited for a criteria-driven select group of patients to increase program capacity, reduce health service utilization, and optimize patients’ experience.^33^ This evidence has facilitated significant global changes in patients’ journey of care to reduce the LoS, and continues to be instrumental in driving improved and timely access to care and optimal outcomes. However, comparisons between European and the US data highlights that LoS after uncomplicated TAVI in Europe remains stubbornly high, with most patients remaining in hospital 5 to 10 days, whereas similar US patients are discharged within 1 to 2 days.^34^, ^35^, ^36^, ^37^ This substantial discrepancy is multifactorial but may be attributed to differences in health care systems. In systems that function on a diagnosis-related flat-rate basis, such as in Germany, the financial obligation is contingent on the duration of the patient’s stay in hospital, whereas the US health care system used a bundled payment model, wherein a predetermined sum is paid per patient, promoting cost minimization and reduced LoS. In consideration of the aforementioned data, a reduction in hospital LoS to 6.4 days through the implementation of Benchmark practices and an additional CVC resulting in 1.2 to 5.2 days in LoS may contribute to significant cost savings in funding models in Europe. This evolution would enable some European regions to align with the patterns of decreased critical care utilization and next-day discharge established in the United States.^38^

LoS is the end product of multiple moving parts of clinical care. In our study, the interplay between changes in practice; the role of the CVC; and the evolving issues of anesthesia strategy, procedure time, and 30-day outcomes emerged as particularly salient to informing practice and future research.

There is significant variation in anesthesia strategies across international regions.^39^ Although Benchmark enabled the increased adoption of minimalist sedation and the associated benefits of nursing care requirements and rapid recovery, the selection of anesthesia strategy is also driven by the important additional considerations such as comorbidities and risk profile, patient preference and procedure-related anxiety, anatomical factors, and vascular access route.^40^, ^41^, ^42^ To ensure that care is tailored to patients’ needs, the CVC may play a pivotal role in preprocedure education to help patients manage expectations and understand the benefits of minimal sedation if appropriate, or support the selection of a more intense anesthesia strategy in careful preprocedure planning as required for patient safety. This context is pertinent to understanding the complex mechanisms of the implementation of Benchmark, the impact of the CVC, and the clinical decisions influencing practices such as anesthesia care.

Although total procedure time and intervention time might be presumed to be predominantly physician-driven, the present data suggest that these parameters may be subject to the indirect influence by CVC-led coordination and the implementation of practice changes. The observed reduction in total procedure and intervention times in our study signals the benefits of scrutiny of all aspects of periprocedure processes. A cohesive approach to team-based quality improvement such as Benchmark places value on the contributions of all members, including nursing and allied professionals. As such, the influence of a CVC on intervention time is challenging to capture. Our study provides early indications that the CVC may play a pivotal role in attending to the multiple factors driving procedural efficiencies. Future research is needed to ascertain if and how the CVC’s unique contributions to early risk stratification, individualized procedure planning, and anticipation of risks may serve to contribute to improved resource utilization.

Lastly, the observed reduction in 30-day major vascular bleeding merits further exploration. Rates of vascular complications continue to decrease due to changes in practice such as the rigorous use of computed tomography imaging in procedure planning and the adoption of ultrasound-guided access and closure, and advances in closure technique and devices.^43^ These practices contribute to a more predictable journey of care and play a pivotal role in safety. Nevertheless, our study suggests that the CVC may play an essential role in ensuring that all details that facilitate the end result of safe hemostasis are systematically attended to. The CVC facilitates a coordinated preprocedure plan that includes early and rigorous evaluation of access site by the medical team, standardized reporting, and clear communication to the procedure team.^44^ Optimally, this process may be further integrated in team-based safety checklists and emergency intervention plans that have a proven impact on procedural success and reduction of complications.^6^

To achieve the end goal of early discharge and sustain a fully optimized TAVI service, programs require leadership and coordination to facilitate change management, meet patient needs, and implement nimble and responsive strategies to support these quality improvement targets. In this context, the recommendations of international guidelines to include a dedicated CVC as an essential member of the multidisciplinary team^12^ may be further validated by our study. Although it is challenging to parse the direct impact of the CVC on the multiple factors that promote the safety of TAVI patients and the full optimization of their care pathway, our findings add novel evidence of the direct or indirect impact of the role on accelerating the adoption of best practices and operational changes within programs and supporting the implementation of efficiencies and patient-centered clinical care.

### The Value Proposition of the Clinical Valve Coordinator

For patients and their families, the CVC offers a single point of contact focusing on a patient-centered communication and shared decision-making, as well as the coordination of procedure planning, discharge, and follow-up.^8^^,^^9^ In the BENCHMARK study, the measurement of patient-reported experiences using a modified version of the Short Assessment of Patient Satisfaction questionnaire^23^ suggested a generally high degree of satisfaction in patients treated with TAVI, irrespective of a CVC’s presence. In a more nuanced analysis, the COORDINATE study reported that the availability of a dedicated CVC significantly increased patients’ overall knowledge of their disease status and planned procedure and their self-efficacy related to the procedure and postprocedure care.^6^ Further research is needed to better ascertain this evidence and inform the development of indicators to measure the impact of CVCs on Patient-Reported Outcomes and Experiences Measures (PROMs and PREMs).

For programs and the multidisciplinary team, additional responsibilities of a CVC include a wide-ranging clinical role, including coordinating or overseeing the TAVI assessment pathway, conducting focused clinical assessments as appropriate (e.g., frailty, function, cognition), sharing their perspectives in heart team reviews and decision-making, attending to the multiple clinical components of procedure planning, and preparing patients and their families for safe early discharge. This seamless approach and coordinated activities contribute to the efficient use of time and resources.^7^^,^^18^^,^^45^

At the hospital level, the CVC may play a central role in strengthening the referral process and communication with primary care providers, community hospitals, and cardiologists. This role is particularly important given the efforts required to address the barriers to the journey of valvular heart disease care, including patients’ awareness of symptoms, detection of disease presence and progression by primary care, referral for echocardiography and diagnosis, and timely referral, assessment pathway, and monitoring before treatment.^3^^,^^46^ As one of the primary contacts for both patients and referrers, the CVC is ideally positioned to remove obstacles for all parties and increase access to care while promoting patient-centered and referrer-targeted processes and efficiencies.

### Consensus and Quality Indicators Needed

The BENCHMARK findings provide some novel insights on the impact of the CVC across 7 diverse countries and health systems, suggesting that the role may be instrumental in implementing contemporary best practices in TAVI programs. This evidence is encouraging but likely insufficient to accelerate the adoption and standardization of the role or to fully leverage its impact. Previous research has reported on the challenges of describing and measuring the direct and indirect effects of CVCs and highlighted the often invisible aspects of clinical coordination.^9^

Professional associations are ideally positioned to build consensus to strengthen CVC role clarity and clinical responsibilities, outline required unique competencies, support professional development, and define essential program infrastructure and organizational integration strategies. Currently, we lack a guiding statement to inform policy to optimize the role of the CVC. Addressing this gap would equip programs in regions where the role remains in its infancy with the required evidence to integrate CVCs in health planning and funding. To this end, the development of quality indicators to measure the sustained impact of the CVC role on access, outcomes, patient experiences, health services, and costs would provide important guidance across international regions.

### Limitations

Although the study demonstrated the beneficial role of the CVC in a real-world TAVI program, several limitations must be acknowledged. First, we acknowledge that the implementation of a CVC role constitutes a complex intervention that is not easily amenable to randomization. The lack of an active control group may have presented a certain selection bias inherent to observational designs. However, the use of propensity score matching significantly mitigated this issue to provide a more accurate estimate of the effect of the CVC. In addition, the observational study design limits any inference of causality. There may be unmeasured confounders that might have influenced the results in spite of the strategies put in place. Another limitation inherent to the observational design and consecutive patient inclusion is the persistence of residual differences in parameters such as BMI and impaired mobility despite propensity score matching. A higher BMI and the absence of impaired mobility have previously been identified as indicators of a favorable outcome for TAVI,^47^^,^^48^ which were present in centers with pre-existing CVC within this study. Therefore, this bias should be considered when evaluating the results. Importantly, the existing intercountry and intracountry variations in patient management and treatment approaches might have impacted outcomes in this large multicountry study. Lastly, although we recognize the importance of integrating PROMs and PREMs in clinical evaluations, we recognize that the exploratory measurement of patient satisfaction in BENCHMARK must be interpreted with caution and merits further research.

## Conclusions

Our findings suggest that the CVC role combined with the implementation of Benchmark best practices is associated with reducing total hospital LoS and critical care resources after TAVI without comprising 30-day safety. Further efforts are required to develop an evaluation framework and impact indicators to build on the findings of the present study. These measures will be essential to support knowledge translation activities to help administrative teams appreciate the value proposition of the CVC across the treatment of valvular heart disease.

## Ethics Statement

This registry complies with the the European Medical Device Regulations and the Declaration of Helsinki. Ethical approval was obtained from the independent ethics review boards at each participating site. All patients provided written informed consent.

## Funding

The study was funded by 10.13039/100006520Edwards Lifesciences (Nyon, Switzerland) and performed under the sponsorship of IPPMed—Institute for Pharmacology and Preventive Medicine GmbH (Cloppenburg, Germany).

## Review Statement

Full responsibility for the editorial process for this manuscript was delegated to Guest Editor Elizabeth M. Perpetua, DNP

## Disclosure Statement

Sandra Lauck received speaker’s fees from Edwards Lifesciences. Francesco Saia received consulting fees and payment for lectures from Edwards Lifesciences, Medtronic, Boston Scientific, and Abbott. Eric Durand received lecture fees from Edwards Lifesciences. Bettina Højberg Kirk received consulting fees and support for the present manuscript from Edwards Lifesciences. Fiona Kelly has an ongoing contract with Edwards Lifesciences; received consulting fees and payments from Edwards Lifesciences for services relating to the Benchmark program; and received payment for presentations and support for attending meetings from Edwards Lifesciences and Medtronic. Douglas F. Muir received honoraria through the BENCHMARK process and payment for presentations and other educational events from Edwards Lifesciences. Gemma McCalmont received consulting fees from Edwards Lifesciences. Mark S. Spence received honoraria for educational activity from Edwards Lifesciences. David Wood received grants and consulting fees from Edwards Lifesciences, Medtronic, and Boston Scientific. Cristóbal A. Urbano Carrillo received consulting fees from Edwards Lifesciences and payments as advisory board member from Medtronic. Vlad Anton Iliescu is a proctor for Edwards Lifesciences. Céline Hee received support for the present manuscript from Edwards Lifesciences and Medtronic. Vincent Auffret received consulting fees from Medtronic and Abbott, and payment for lectures/presentations or educational events from Edwards Lifesciences. Lluis Asmarats received consulting fees from Edwards Lifesciences. The institution of Carlo Di Mario received research funding form Edwards Lifesciences. Andreas Schober received educational/research grants, speakers fees, and meeting & travel support from Edward Lifesciences and Medtronic. Luis Nombela-Franco is a proctor for Edwards Lifesciences and received consulting fees and honoraria for lectures, presentations or educational events from Edward Lifesciences, Abbott, and Boston Scientific. Nikos Werner received honoraria for lectures/presentations and support for attending meetings from Edwards Lifesciences, Medtronic, and Meril; and participates on a Data Safety Monitoring Board or Advisory Board from Boston Scientific, Shockwave, and Meril. The institution of Nicolas Meneveau received grants or contracts from Medtronic and Boston Scientific; he himself received consulting fees from Abbott, Boston Scientific, Edwards Lifesciences, and Terumo, payment for lectures/presentations or educational events from Astra Zeneca, and support for attending meetings from Servier. Felix Mahfoud received grants from Deutsche Forschungsgemeinschaft (SFB TRR219, Project-ID 322900939) and Deutsche Herzstiftung; consulting fees and honoraria for lectures, presentations, or educational events from Ablative Solutions, Astra-Zeneca, Inari, Medtronic, Merck, Novartis, Philips, and ReCor Meidcal; and is an European Society of Cardiology Board Member. Tim Seidler received payment for lectures/presentations or educational events from Astra Zeneca, Boehringer Ingelheim, Bristol Myers Squibb, Corvia, Edwards Lifesciences, Medtronic, Myocardia, Novartis, and Pfizer; support for attending meetings from Teleflex, Corvia, Edwards Lifesciences, Abbott, Medtronic, and Bristol Myers Squibb; and participates on a Data Safety Monitoring Board or Advisory Board from Bristol Myers Squibb. Florian Leuschner received honoraria for lectures form Edwards Lifesciences and Medtronic. Elmar Kuhn received consulting fees and payment for proctoring from Medtronic and Abbott. Radka Rakova, Wilbert Wesse, and Jana Kurucova are employees of Edward Lifesciences. Peter Bramlage is representing the sponsor IPPMed who received funding form Edwards Lifesciences to his institution for the present manuscript, study related activities, consulting fees, and support for attending meetings. Violetta Hachaturyan, Claudia M. Lüske, and Marie Zielinski are employees of IPPMed and received funding from Edwards Lifesciences to the institution for conducting the registry. Derk Frank received consulting fees and payment for lectures/presentations from Edwards Lifesciences; he or his personal surrounding also received support for the present manuscript. The reported conflicts of interest did not influence the analysis or interpretation of the study results.

The other authors had no conflicts to declare.
